# Development and validation of a radiomic nomogram based on pretherapy dual-energy CT for distinguishing adenocarcinoma from squamous cell carcinoma of the lung

**DOI:** 10.3389/fonc.2022.949111

**Published:** 2022-11-23

**Authors:** Zhiyong Chen, Li Yi, Zhiwei Peng, Jianzhong Zhou, Zhaotao Zhang, Yahong Tao, Ze Lin, Anjing He, Mengni Jin, Minjing Zuo

**Affiliations:** ^1^Department of Radiology, The Second Affiliated Hospital of Nanchang University, Nanchang, China; ^2^Department of Radiology, The Quzhou City People’s Hospital, Quzhou, Zhejiang, China

**Keywords:** dual-energy CT, dual-energy CT quantitative parameters, radiomics, lung adenocarcinoma, lung squamous cell carcinoma

## Abstract

**Objective:**

Based on pretherapy dual-energy computed tomography (DECT) images, we developed and validated a nomogram combined with clinical parameters and radiomic features to predict the pathologic subtypes of non-small cell lung cancer (NSCLC) — adenocarcinoma (ADC) and squamous cell carcinoma (SCC).

**Methods:**

A total of 129 pathologically confirmed NSCLC patients treated at the Second Affiliated Hospital of Nanchang University from October 2017 to October 2021 were retrospectively analyzed. Patients were randomly divided in a ratio of 7:3 (n=90) into training and validation cohorts (n=39). Patients’ pretherapy clinical parameters were recorded. Radiomics features of the primary lesion were extracted from two sets of monoenergetic images (40 keV and 100 keV) in arterial phases (AP) and venous phases (VP). Features were selected successively through the intra-class correlation coefficient (ICC) and the least absolute shrinkage and selection operator (LASSO). Multivariate logistic regression analysis was then performed to establish predictive models. The prediction performance between models was evaluated and compared using the receiver operating characteristic (ROC) curve, DeLong test, and Akaike information criterion (AIC). A nomogram was developed based on the model with the best predictive performance to evaluate its calibration and clinical utility.

**Results:**

A total of 87 ADC and 42 SCC patients were enrolled in this study. Among the five constructed models, the integrative model (AUC: Model 4 = 0.92, Model 5 = 0.93) combining clinical parameters and radiomic features had a higher AUC than the individual clinical models or radiomic models (AUC: Model 1 = 0.84, Model 2 = 0.79, Model 3 = 0.84). The combined clinical-venous phase radiomics model had the best predictive performance, goodness of fit, and parsimony; the area under the ROC curve (AUC) of the training and validation cohorts was 0.93 and 0.90, respectively, and the AIC value was 60.16. Then, this model was visualized as a nomogram. The calibration curves demonstrated it’s good calibration, and decision curve analysis (DCA) proved its clinical utility.

**Conclusion:**

The combined clinical-radiomics model based on pretherapy DECT showed good performance in distinguishing ADC and SCC of the lung. The nomogram constructed based on the best-performing combined clinical-venous phase radiomics model provides a relatively accurate, convenient and noninvasive method for predicting the pathological subtypes of ADC and SCC in NSCLC.

## 1 Introduction

Lung cancer is the second most common cancer worldwide and the leading cause of cancer death (1). Non-small cell lung cancer (NSCLC) accounts for approximately 85% of lung cancers, with adenocarcinoma (ADC) and squamous cell carcinoma (SCC) being the most common subtypes (2, 3). In recent years, the prognosis of some lung cancer patients has improved thanks to the rapid development of individualized medicine and precise therapy, such as targeted therapies and immunotherapy (4–6). However, different pathological subtypes have distinct phenotypic and biological characteristics, which directly affect clinical treatment and outcomes (6–8). For example, bevacizumab has good effects in the treatment of lung adenocarcinoma, but it may lead to a lung squamous cell carcinoma patient bleeding profusely (9). Therefore, it is important to accurately predict pathological subtypes before treatment to establish better therapeutic strategies for NSCLC.

Currently, invasive biopsy for histological confirmation is usually performed before the treatment of NSCLC (9, 10). However, it is difficult to obtain a biopsy for several reasons. First, lung cancer is a heterogeneous tumor, and the tissue obtained from the biopsy of the lung tumor may contain only a few tumor cells and may not reflect the complete biological information (5, 9). Then, tumor samples are difficult to obtain in some patients, ang biopsy is contraindicated, and so on. In addition, biopsy may also increase the potential risk of cancer transmission (11). Therefore, it is necessary to develop a reliable, non-invasive, safe and economical approach to help pretherapy predict the pathological subtypes in NSCLC for treatment decision-making and prognosis estimation in NSCLC patients.

Dual-energy computed tomography (DECT) is a new technology in the field of CT imaging in recent years. It not only shows the morphological features of tumors, but also provides extensive quantitative information (12). Many studies have used DECT for tumor diagnosis and prediction. Zhang et al. (13) found that quantitative parameters based on venous phase DECT, including iodine concentration (IC), normalized iodine concentration (NIC), and slope of the curve (λHU), can effectively distinguish ADC and SCC of the lung. Radiomics analyzes medical images in an automated high-throughput manner and aims to extract quantitative and reproducible tumor information that the human eye cannot distinguish, quantify tumor heterogeneity, and monitor tumor development, progression, and even prognosis (14–17). Many studies have explored the role of radiomics in the pathological classification of NSCLC. Zhu et al. (18) enrolled 129 NSCLC patients for retrospective studies, and the LASSO regression model was constructed by screening 5 radiomic features. The result was that the radiomic features could be used as a diagnostic factor to distinguish the histological subtypes of NSCLC.

To further explore the additional value of the DECT image, some studies combine DECT with radiomics. Liu et al. (19) built and evaluated a pretherapy dual-energy CT-based clinical-radiomics model that can effectively predict the clinical response to systemic chemotherapy in patients with advanced gastric cancer (AGC). However, to our knowledge, the application and potential advantages of DECT-based radiomics in predicting the pathological subtypes of NSCLC have not been explored. Theoretically, DECT contains more information than single-energy CT. Radiomic analysis of DECT images may extract more features relevant to tumor heterogeneity and biology.

Therefore, the aim of this study was to establish an independent predictive model for predicting the pathological subtypes of NSCLC by combining clinical parameters and DECT-based radiomic features. In addition, we provide a visually quantitative nomogram in clinical practice, as an additional predictive method for patients who cannot obtain pathological subtypes before treatment.

## 2 Materials and methods

### 2.1 Patients

Eligible patients with NSCLC treated at the Second Affiliated Hospital of Nanchang University between October 2017 and October 2021 were retrospectively analyzed. This single-center retrospective study was approved by the Ethics Committee of Second Affiliated Hospital of Nanchang University (Ethics Number: 2017061), and the requirement of informed consent was exempted due to the retrospective study design. The inclusion criteria were as follows: 1) all patients had standard DECT plain scan and enhanced scan images; 2) all lesions were examined by DECT within two weeks, and pathological results were confirmed by puncture biopsy, fiberoptic bronchoscopy or surgical resection; 3) lesion diameter >10 mm, and the boundary was clear; and 4) all patients had detailed clinical data, including age, sex, smoking history, etc. Exclusion criteria included the following: 1) patients who have been or are being treated for oncological disease; 2) dense metal or implants interference in the scanning area; and 3) patients who cannot cooperate during scanning and who experience respiratory motion artifacts.

**Figure 1:**
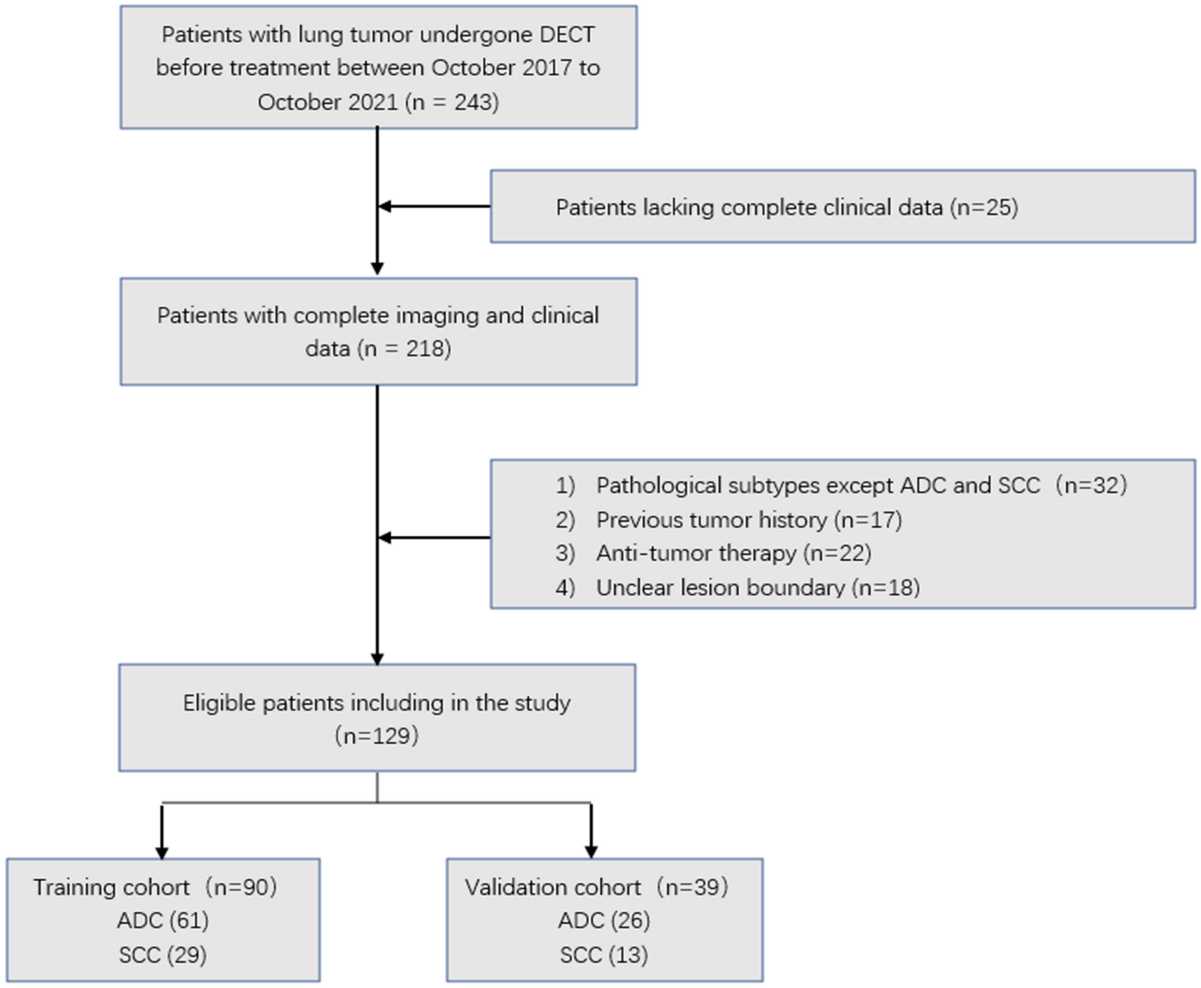
Flow chart showing the patient selection and exclusion.

The patient recruitment process is presented in **Figure 1**. A total of 129 patients were randomly divided at a ratio of 7:3 into training and validation cohorts. The training cohort consisted of 90 patients (ADC 61, SCC 29), whereas the validation cohort consisted of 39 patients (ADC 26, SCC 13).

### 2.2 Clinical features

The following pretherapy clinical features of each patient were recorded from the medical system: age, sex, smoking status (never, ever/always), carcinoembryonic antigen (CEA) level, and distant metastasis (with/without).

### 2.3 Dual-energy CT image acquisition

The patient was in the supine position. After breath holding at the end of inhalation, the dual-energy plain scan and dual-energy enhanced scan in AP and VP were performed from the thoracic inlet to the bottom of the lung.

CT scans were performed in DE mode on a second-generation dual-source CT scanner (SOMATOM Definition FLASH, Siemens Healthcare, Germany). After unenhanced CT was performed, 350 mg I/mL of nonionic iodinated contrast agent (Ioversol) at a dose of 1.2 mL/kg weight was injected into an antecubital vein together with 20 mL of saline at rates of 3 mL/s and 4 mL/s, respectively. AP and VP dual-energy contrast-enhanced CT images were obtained after post-injection delays of 30 and 60 s, respectively. The scan parameters for the DECT mode were summarized as follows. The tube voltages of A and B were set at 100 kVp and 140 kVp, respectively, with a real-time adjustable variable tube current. Collimation was 128 × 0.6 mm; rotation speed was 0.28 s/r; gantry rotation was 330 ms; slice thickness was 5 mm. Finally, 100 kVp and 140 kVp images were acquired in the arterial and venous phases, respectively, and 120 kVp equivalent mixed images were generated (linear fusion coefficient, 0.4). These images were reconstructed with a slice thickness of 1 mm and an interval of 1 mm using iterative reconstruction software (SAFIRE, Siemens Healthcare, Germany).

### 2.4 Dual energy-CT image analysis

CT Semantic Feature Acquisition: Two radiologists (with five and fifteen years of experience in diagnostic thoracic imaging), blinded to the patient’s pathologic data, viewed and analyzed the 120 kVp equivalent hybrid images and obtained CT semantic features of each lesion. Six CT semantic features for each mass were included (1): spiculation sign (2), lobulation sign (3), null vacuole sign (4), tumor location (central/peripheral type) (5), pleural effusion on the tumor side (yes/no), and (6) pericardial effusion (yes/no). If any disagreements arose, final consensus was reached through group discussions.

DECT Quantitative Parameter Acquisition: Data from AP and VP DECT were loaded and postprocessed using specific software (Siemens Healthcare, Germany). The iodine diagram was obtained by the Liver VNC program. Manually, the region of interest (ROI) was drawn as large as possible on the solid part of the primary lesion, avoiding tumor margins, necrosis, cavities, calcifications and large vessels. Then, the iodine concentration (IC, mean value, units of 100 μg/ml) of the lesion was recorded in the ROI. Simultaneously, ROIs were placed in the same slice to obtain the ICs of the aorta. Finally, the normalized iodine concentration (NIC) was calculated according to the following formula: NIC = IC (lesion)/IC (artery). Then, through the Monogenetic program, the CT values of 40 keV and 100 keV single energy images of the solid part of the lesion were recorded. The slope of the spectrum attenuation curves (λHU) was calculated using the following formula: λHU=((CT40KeV-CT100keV)/60). All data were measured three times and averaged.

### 2.5 Radiomic analysis

#### 2.5.1 Tumor segmentation

The 40-keV and 100-keV monoenergetic images (NIFTI format) reconstructed in AP and VP were imported into the open source software ITK-snap (version 3.8.0, University of Pennsylvania, USA, http://www.itksnap.org). A radiologist (with five years of experience in diagnostic thoracic imaging) performed semi-automatic or manual combined semi-automatic layer-by-layer segmentation of the lung window.

#### 2.5.2 Feature extraction

Artificial Intelligence Kit software (A.K. Software; GE Healthcare, China) was used to extract the radiomics features from each ROI. A total of 107 features were extracted including first-order statistical features, shape features, and texture features. In addition, the software provides a variety of options to standardize image preprocessing before feature extraction. The extracted features were reproducible and based on the benchmarks of the image biomarker standardization initiative (IBSI).

#### 2.5.3 Feature selection

To assess segmentation variability, 20 patients were randomly selected and re-segmented after one month by the same two radiologists. The inter- and intra-observer reproducibility of tumor segmentation was assessed by intraclass correlation coefficients (ICCs). The features with an ICC greater than 0.75 are defined as having good repeatability. After selecting the repeatable features based on ICC, the LASSO algorithm was applied to select the most useful predictive features in the training cohort.

#### 2.5.4 Radiomics model establishment

Radiomics models were established by multivariable logistic regression analysis of radiomic features selected in the images from AP and VP DECT. Radiomic signatures, also called the radiomic score (Rad-score), were calculated separately for the training and validation cohorts in the AP and VP *via* a linear combination of selected features weighted by their respective coefficients in the model.

### 2.6 Clinical model and nomogram establishment

Clinical features, CT semantic features, and DECT quantification parameters are collectively referred to as clinical parameters in this study.

Univariate analysis was performed for candidate clinical parameters. The significant variables (p value < 0.05) in the univariable analysis were then introduced into stepwise logistic regression analyses. The independent clinical predictors were determined and the clinical model was established. Then, the selected clinical predictors were combined with the radiomic signatures of the arterial and venous phases to establish two combination models. To visualize the prediction results of the model for ADC and SCC, the nomogram was developed based on the model with the best performance.

### 2.7 Evaluation and comparison of model performance

Evaluation of the model included discrimination, calibration, and clinical utility. Receiver operating characteristic (ROC) curve analysis was used to evaluate the predictive performance of each model. The Delong test was used to compare the difference in the area under the curve (AUC) between different models. The Akaike information criterion (AIC) is used to compare the goodness of fit and parsimony between models. Calibration curves were constructed to describe calibration performance based on the agreement between predicted and actual response probabilities. Decision curve analysis (DCA) was used to determine the value of the predictive model for clinical application and to determine the net benefit to patients at each threshold probability.

### 2.8 Statistical analysis

IBM SPSS 25.0 (IBM, Armonk, NY, USA) software was used for statistical analysis of clinical parameters: Normality of distribution of continuous variables was tested using a Kolmogorov–Smirnov test; independent samples t-test (or Mann-Whitney U-test) for continuous variables and chi-square test for categorical variables.

Other statistical analyses were conducted with R (version 4.1.2, http://www.r-project.org) software. The “MASS” package was used for stepwise logistic regression to further filter clinical features. The “glmnet” package was used for lasso logistic regression to filter radiomic features and multiple logistic regression to build models. The “pROC” package was used for plotting ROC curves and calculating AUC values and related indicators. And the “rms” package was used for drawing nomograms and calibration curves. The Delong test was used for comparison between models, and the Akaike information criterion (AIC) was used for model ranking and selection. Two-sided p values < 0.05 indicate statistical significance.

## 3 Results

### 3.1 Clinical parameters

A total of 129 NSCLC patients, including 87 ADC patients and 42 SCC patients, were enrolled in this study. After univariate analysis, eight clinical parameters, including age, sex, smoking status, spiculation sign, tumor location (central/peripheral type), distant metastasis (with/without), NIC and λ HU in the VP, were significantly associated with the pathological subtypes of NSCLC (p < 0.05; the results of univariate analysis of patients’ clinical parameters are shown in **Table 1**). Subsequently, three of these parameters (sex, distant metastasis, and NIC in the VP) were selected using stepwise logistic analysis to form the clinical model (related data in **eTable 1** in the **Supplementary Materials**).

**Table 1:** Clinical parameters of patients.

Variables	Training cohort (n =90)		P	Validation cohort (n = 39)		P
	ADC (n=61)	SCC (n=29)		ADC (n=26)	SCC (n=13)	
Age (year)	62.03 ± 9.06	66.27 ± 8.97	0.040	60.08 ± 9.96	63.62 ± 9.81	0.300
Gender			<0.001*			0.022*
Male	30	28		11	12	
Female	31	1		15	1	
Smoking			<0.001*			0.029*
Never	15	15		7	9	
Ever/Always	46	10		19	4	
Spiculation			0.004*			0.307
Yes	48	13		16	5	
No	13	14		10	8	
lobulation			0.930			0.397
Yes	53	25		24	10	
No	8	4		2	3	
null Vacuole			0.628			0.687
Yes	12	7		7	2	
No	49	22		19	11	
tumor location			0.030*			0.687
Peripheral	53	25		24	10	
Central	8	4		2	3	
pleural effusion on the tumor side			0.738			0.852
Yes	5	3		2	2	
No	56	26		24	11	
pericardial effusion			0.274			0.608
Yes	5	0		1	1	
No	56	29		25	12	
distant metastasis			0.016*			0.420
Yes	21	3		7	2	
No	40	26		19	11	
CEA(ug/L)	3.18 (1.76,9.90)	2.60 (2.02,5.20)	0.610	2.33 (1.63,5.50)	3.28 (1.37,4.37)	0.532
NIC_AP_	0.09 (0.04,0.18)	0.08 (0.01,0.14)	0.223	0.12 (0.05,0.20)	0.05 (0.03,0.12)	0.136
λHU_AP_	1.39 ± 1.03	1.14 ± 1.02	0.267	1.86 ± 1.40	1.01 ± 0.65	0.044*
NIC_VP_	0.28 (0.16,0.46)	0.19 (0.08,0.33)	0.019*	0.37 (0.21,0.47)	0.17 (0.03,0.23)	0.003*
λHU_VP_	1.88 ± 1.07	1.21 ± 0.82	0.004*	2.17 ± 1.13	0.96 ± 0.69	0.001*

Data are the proportion of sample size, mean value ± SD or median (interquartile range). P values were the results of univariate analysis of each parameter, *p < 0.05.

AP, arterial phase; VP, venous phase.

### 3.2 Radiomic features selection and radiomic signature building

**Figure 2:**
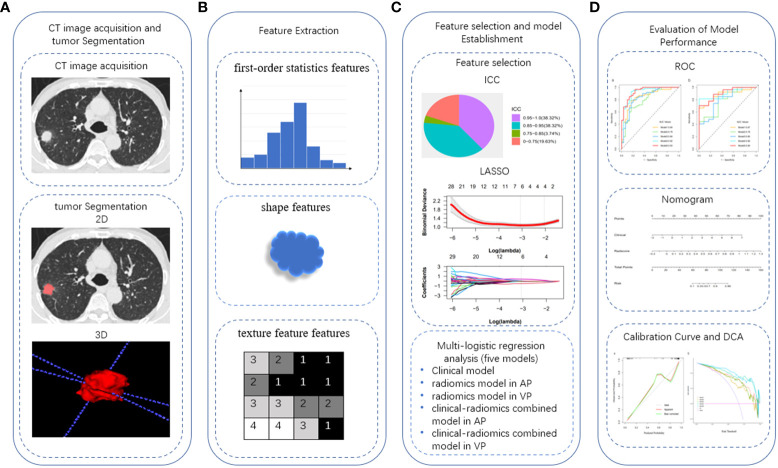
Work flow of tumor segmentation, feature extraction and signature building. ICC, intra-class correlation coefficient; LASSO, the least absolute shrinkage and selection operator; ROC, receiver operating characteristic; DCA, decision curve analysis.

The workflow of tumor segmentation, feature extraction and selection, model establishment and evaluation is illustrated in **Figure 2**. A total of 107 features were extracted from the reconstructed 40 keV and 100 keV monoenergetic images from AP and VP DECT for each patient, respectively. Excluding features with low reproducibility according to ICC (intra- and inter-observer ICC <0.75, ICC results are shown in **eTable 2** in the **Supplementary Materials**). Thus, the numbers of 40 keV and 100 keV in the AP (AP40 keV, AP100 keV), and 40 keV and 100 keV in the VP (VP40 keV, VP100 keV) features were reduced to 76, 78, 86 and 84 respectively. Then, the LASSO algorithm was used to exclude redundant features. This left 2, 3, 5, and 5 features at AP 40 keV, AP 100 keV, VP 40 keV, and VP 100 keV respectively (**eTable 3** in the **Supplementary Materials**). Finally, the five features selected from 40-keV and 100-keV DECT images in AP were combined, and the radiomic signature based on AP (rad-score AP) was established by multivariate logistic regression analysis in the training cohort. The same method was used to establish the radiomic signature based on VP (rad-scoreVP). The radiomic score calculation formula is presented in **eTable 1** in the **Supplementary Materials**.

### 3.3 Prediction model establishment and evaluation of model performance

All models were established by multivariate logistic regression analysis.

Clinical model: The clinical model (Model 1) consisted of three clinical parameters (sex, distant metastasis, and NIC in the VP). The AUCs of the training and validation cohorts were 0.84 (95% CI 0.75-0.93) and 0.87 (95% CI 0.77-0.98) respectively.

Radiomics model: The AUCs for the radiomics model in AP (Model 2) and the radiomics model in VP (Model 3) in the training cohort were 0.79 (95% CI 0.69-0.89) and 0.84 (95% CI 0.75-0.93), respectively; in the validation cohort, they were 0.78 (95% CI 0.63-0.93) and 0.80 (95% CI 0.64-0.95), respectively. Compared with AP, the AUC of the VP radiomics model was higher, but there was no significant difference between the 2 AUCs (Delong test, P = 0.067).

Combined model: The combined clinical-arterial phase radiomics model (Model 4) and the combined clinical-venous phase radiomics model (Model 5) were established by combining the clinical parameters with the radiomic features of AP and VP, respectively. The AUC values were 0.92 (0.86-0.98) and 0.93 (0.88-0.98) in the training cohort and 0.90 (0.81-0.99) and 0.90 (0.81-0.99) in the validation cohort.

**Figure 3:**
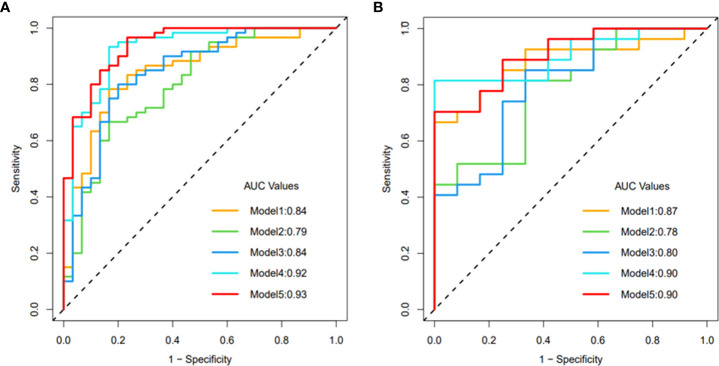
ROC curve of model 1-5: **(A)** training cohort, **(B)** validation cohort.

The results showed that the predictive performance of the combined model was significantly higher than that of the single radiomic or clinical model (DeLong test, p > 0.05 for each comparison). The combined clinical-venous phase radiomics model (Model 5) had the best predictive performance (AUC: training cohort 0.93, validation cohort 0.90), but there was no significant difference in AUC between Model 5 and Model 4(DeLong test, p=0.384). In addition, the Akaike information criterion (AIC) was introduced to evaluate the goodness and parsimony of fit of the model, and Model 5 achieved the lowest AIC value at 60.16 among all prediction models. Based on the overall consideration of ROC curves and AIC, Model 5 was proven to have the best predictive performance, good goodness of fit and parsimony. The ROC curves, detailed performance and AIC values of the five models are illustrated in **Figure 3** and **Table 2**. The result of the DeLong test is given in **Table 3**.

**Table 2:** Prediction performance of model 1-5.

Cohort	Model	AUC (95%CI)	SEN	SPE	ACC	PPV	NPV	AIC
Training cohort	Model 1	0.84(0.75-0.93)	0.78	0.83	0.80	0.93	0.66	88.87
	Model 2	0.79(0.69-0.89)	0.66	0.83	0.72	0.88	0.65	90.81
	Model 3	0.84(0.75-0.93)	0.80	0.80	0.79	0.88	0.66	83.36
	Model 4	0.92(0.86-0.98)	0.93	0.83	0.90	0.91	0.86	64.06
	Model 5	0.93(0.88-0.98)	0.96	0.76	0.90	0.89	0.92	60.14
Validation cohort	Model 1	0.87 (0.77-0.98)	0.66	1.00	0.82	1.00	0.63	—
	Model 2	0.78 (0.63-0.93)	0.81	0.66	0.76	0.84	0.61	—
	Model 3	0.80(0.64-0.95)	0.85	0.66	0.79	0.85	0.66	—
	Model 4	0.90(0.81-0.99)	0.81	1.00	0.87	1.00	0.70	—
	Model 5	0.91(0.81-0.99)	0.70	1.00	0.79	1.00	0.60	—

AUC, area under the curve; CI, confidence interval; SEN, sensitivity; SPE, specificity; ACC, accuracy; PPV, positive predictive value; NPV, negative predictive value; AIC, Akaike information criterion.

**Table 3:** Delong test between models 1-5.

Model 1	1				
Model 2	0.500	1			
Model 3	0.945	0.067	1		
Model 4	0.035*	0.003*	0.036*	1	
Model 5	0.016*	0.001*	0.009*	0.348	1
	Model 1	Model 2	Model 3	Model 4	Model 5

*P<0.05.

### 3.4 Development and performance evaluation of the nomogram

Based on the above results, Model 5 with the best prediction efficiency was selected and visualized as a nomogram for individualized patient prediction. Multivariate logistic regression analysis showed that the clinical signature (odds ratio (OR) = 1.11; 95% CI, 1.07 to 1.16; p < 0.001) and radiomic signature (odds ratio (OR) = 2.21; 95% CI, 1.68 to 2.90; p < 0.001) represented independent predictors in the nomogram (**eTable 4** in the **Supplementary Materials**).

**Figure 4:**
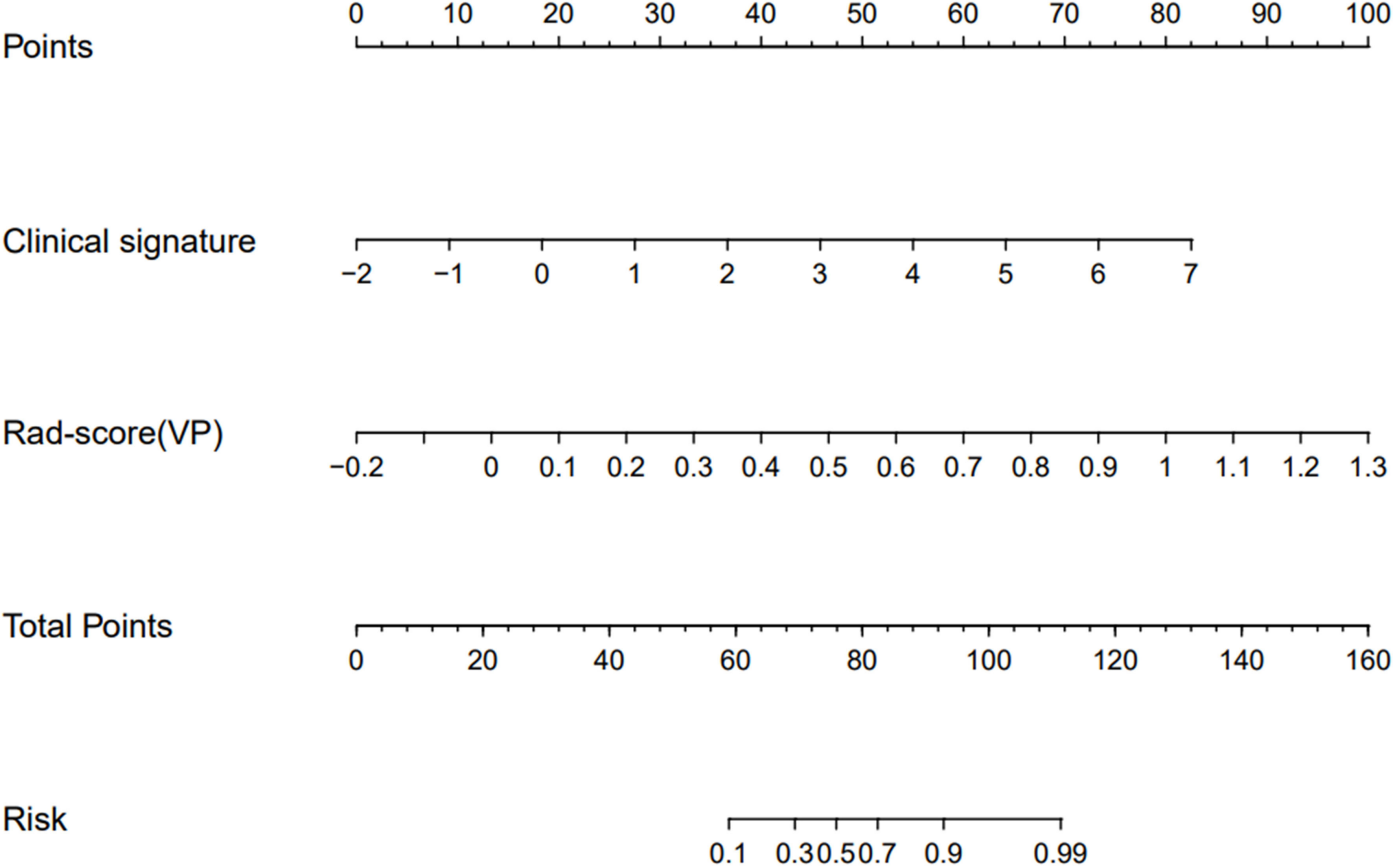
Nomogram based on the clinical signature and radiomic signature in venous phase to predict the pathological subtypes of NSCLC patients.

As shown in the nomogram (**Figure 4**), the radiological signature accounted for the largest proportion compared with the clinical signature, making it the most important biomarker for distinguishing ADC from SCC. In clinical practice, based on the obtained features, the clinical signature and radiomic signature can be calculated using the formula. Then, the probability of the predictive variable was converted into a fraction corresponding to the first scale “point” at the top of the nomogram. After adding up the corresponding prediction probability, the risk of ADC was at the bottom of the nomogram.

**Figure 5:**
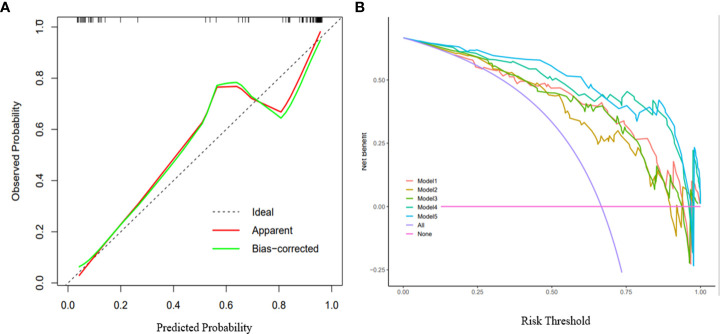
Calibration curves and decision curve analysis of the nomogram. **(A)** Calibration curves of the nomogram: The X-axis represented the predicted probability estimated by nomogram, whereas the Y-axis represented the actual observed rates. the red line represented the apparent prediction of nomogram, the green line represented the fitting line after bias-corrected. the dashed line represented the ideal estimation. Calibration curves showed the actual probability is relatively close to the prediction of nomogram. **(B)** Decision curve analysis for the models 1-5: The X-axis represented the threshold probability that was where the expected benefit of treatment was equal to the expected benefit of avoiding treatment. The Y-axis represented the net benefit. The purple line represents the hypothesis that all patients are ADC, and the red line represents the hypothesis that all patients are SCC. Other curves are shown in the figure, representing models 1-5 in turn.

The calibration curves (**Figure 5A**) of the nomogram demonstrated good agreement between the nomogram prediction and the actual observation. A nonsignificant difference in the accompanied Hosmer–Lemeshow test (p=0.384) indicated that the nomogram was adequately calibrated without departure from the ideal fit. DCAs (**Figure 5B**) were used to evaluate the clinical utility of the five predictive models by calculating the net benefit at various probability thresholds. According to the decision curves, Model 5 was the most reliable clinical treatment tool for predicting pathologic subtypes in NSCLC when the probability threshold was above 0.25 in a patient’s or physician’s clinical decision.

## 4 Discussion

In this study, we successfully developed and validated a combined clinical-radiomics model based on DECT, which has excellent performance in noninvasively stratifying the pathological subtypes of NSCLC patients. Furthermore, we visualized this model as a nomogram and demonstrated the excellent performance of the nomogram by high AUC and low AIC. DCAs indicated that the nomogram is a reliable clinical treatment decision support tool for personalized prediction of the pathological subtypes of NSCLC patients.

Different pathological subtypes lead to different clinical treatment strategies and prognoses for NSCLC patients (20–22). Dual-energy imaging improves image quality to some degree, expands the capabilities of traditional CT, and has the potential to improve lesion detection and characterization (23–25).Several previous studies have combined DECT with radiomics or texture analysis (26–29). However, most feature extractions are based on virtual monoenergetic, 120 kV equivalent hybrid images or iodine images. Recently, some researchers have demonstrated in their studies that radiomic models based on multi-energy images can more effectively support the diagnosis and prediction of tumors compared with clinical and monoenergetic models As demonstrated by Liu et al. (19), the radiomics model based on multi-energy images can better predict the clinical response of systemic chemotherapy in advanced gastric cancer (AGC) compared to clinical and monoenergetic models. This study extracts the radiomics features from DECT multi-energy images and jointly constructs the model, which proves that the image radiomics features extracted from DECT multi-energy images can reflect the heterogeneity of NSCLC. Radiomics may serve as a promising technique to predict the pathological subtypes of NSCLC.

Among the clinical features selected in the combined model, SCC is more common in males, and this sex difference among NSCLC patients has been widely reported (30, 31). Distant metastasis is more common in patients with ADC than in those with SCC, which is also consistent with the biological characteristics that lung adenocarcinoma is prone to early hematogenous metastasis. In addition, the results of univariate and multivariate analyses in this study showed that NICVP was also an important clinical predictor. In enhanced DECT, IC represents iodine deposition in tissue. The quantification of IC can reflect the microvessel density (MVD) and perfusion of the tumor (32–34). In this study, the NIC of adenocarcinoma was higher than that of squamous cell carcinoma, indicating that the MVD of adenocarcinoma was greater, which is consistent with the results of previous pathological studies (35, 36). Moreover, this result was significantly different in the venous phase but not in the arterial phase. This may be due to the different microvessel densities and vascular permeabilities of different subtypes of tumors, resulting in different times of iodine contrast agent penetration into the intercellular space. This is consistent with the results of a previous study by Zhang et al (13).

In terms of image selection, in DECT scanning, due to the high X-ray attenuation at lower energy levels, when the photon energy gradually decreases from 100 keV to 40 keV, the contrast of the iodized structure gradually increases, but it is also accompanied by an increase in image noise at lower energy levels (37). Therefore, we selected 120 kV equivalent hybrid images with both high contrast and low background noise in evaluating the semantic features of CT lesions (38). In terms of monoenergetic selection for radiomics model establishment, we chose a 100 KeV image with fine detail but low contrast, and a 40 KeV image with higher contrast but more noise. As a result, the AUC of the multi-energy image-based radiomics model was 0.79 and 0.84 in the AP and VP, respectively. This shows that the combination of different energy images can deeply mine tumor information and effectively distinguish ADC from SCC.

In terms of radiomic features, three types of radiomic features were extracted: 1) “First-order statistics: Energy”, describing the overall density of the tumor volume; 2) “Shape: Compactness”, quantifying the compactness of the tumor volume relative to that of a sphere (i.e., the most compact shape); and 3) “texture features: spatial arrangement relationship between voxel gray levels”, describing intra-tumor heterogeneity (39, 40). Among the features we selected, compared with the AP, the VP increased the first-order statistics features (original first-order kurtosis, original first-order skewness), and the first-order statistics features reflected the overall density of the tumor. We believe that this is also related to the different MVD and vascular permeability of different subtypes of tumors, which may also lead to the slightly lower AUC in the AP-based model than VP.

Our study had some limitations. First, it is a retrospective study at a single center, which may lead to patient selection bias, and the number of samples is limited. Future plans include collaboration with other dual-energy CT centers to reduce bias and expand the sample size. Second, the previous radiomics model developed by He et al. (41) for the differential diagnosis of solitary pulmonary nodules showed better differential diagnosis performance based on the radiomics features of plain CT images than those of contrast-enhanced CT images. This study lacks a radiomics model based on plain CT. In the future, it is expected to build a radiomics model based on DECT plain scans to mine tumor features more comprehensively and improve the performance of the model. Finally, semi-automatic or semiautomatic and manual combined segmentation of lesions is time-consuming and variable. In the future, it is expected to combine radiomics with machine learning or deep learning to create better models.

## 5 Conclusion

In this study, we developed and validated a combined clinical-radiomics model based on pretherapy DECT to reliably predict ADC and SCC. Compared with the traditional single clinical model, the combined model significantly improved the prediction performance of ADC and SCC. The combined clinical-venous phase radiomics model was visualized as a nomogram, which could provide a relatively accurate, convenient, and noninvasive method for the individualized discrimination of ADC from SCC in NSCLC patients and assist in clinical decision-making.

## Data availability statement

The raw data supporting the conclusions of this article will be made available by the authors, without undue reservation.

## Ethics statement

The studies involving human participants were reviewed and approved by The Second Affiliated Hospital of Nanchang University Medical Research Ethics Committee. Written informed consent for participation was not required for this study in accordance with the national legislation and the institutional requirements.

## Author contributions

MZ and ZC designed the study. ZC and JZ collected and classified the data. ZC, LY, ZZ, and ZP did the statistical analysis. YT, AH, ZL, and MJ made substantial revisions to the manuscript. All authors contributed to the article and approved the submitted version.

## Funding

This study was supported by Jiangxi Provincial Education Department Key Projects (grant number GJJ200106).

## Acknowledgments

We thank the Patient for his kind cooperation.

## Conflict of interest

The authors declare that the research was conducted in the absence of any commercial or financial relationships that could be construed as a potential conflict of interest.

## Publisher’s note

All claims expressed in this article are solely those of the authors and do not necessarily represent those of their affiliated organizations, or those of the publisher, the editors and the reviewers. Any product that may be evaluated in this article, or claim that may be made by its manufacturer, is not guaranteed or endorsed by the publisher.
