# Supplementary material for: Development and validation of a radiomic nomogram based on pretherapy dual-energy CT for distinguishing adenocarcinoma from squamous cell carcinoma of the lung
Source: Front Oncol. 2022 Nov 23;12:949111. doi: 10.3389/fonc.2022.949111 (PMC9727167; doi:10.3389/fonc.2022.949111)
Supplement: Supplementary file 1 [file Table_1.docx]

**eTable 1.** Essential radiomic features and formula composition

| Model | Intercept/Feature Name | Regression coefficient |
| --- | --- | --- |
| Clinical signature | Intercept=1.4150 | β |
|  | gender | -2.7832 |
|  | distant metastasis | 2.1953 |
|  | NIC(VP) | 3.5692 |
| rad-score (AP) | Intercept=0.6666 | β |
| 40keV | original_shape_Maximum2DDiameterRow_40 | -0.1665 |
|  | original_shape_Maximum2DDiameterSlice_40 | 0.0093 |
| 100keV | original_gldm_LargeDependenceEmphasis_100 | -0.2629 |
|  | original_gldm_LargeDependenceHighGrayLevelEmphasis_100 | -0.0804 |
|  | original_glrlm_LongRunEmphasis_100 | 0.2125 |
| rad-score (VP) | Intercept=0.6667 | β |
| 40keV | original_firstorder_Kurtosis_40 | -0.0366 |
|  | original_glcm_ClusterShade_40 | 0.0692 |
|  | original_glszm_GrayLevelNonUniformity_40 | -0.1982 |
|  | original_glszm_SizeZoneNonUniformity_40 | -0.2610 |
|  | original_shape_SurfaceVolumeRatio_40 | -0.0460 |
| 100keV | original_firstorder_Skewness_100 | 0.0530 |
|  | original_gldm_LargeDependenceHighGrayLevelEmphasis_100 | -0.0835 |
|  | original_glrlm_RunLengthNonUniformity_100 | 0.3827 |
|  | original_glrlm_ShortRunEmphasis_100 | 0.0999 |
|  | original_shape_MajorAxisLength_100 | 0.0054 |

Abbreviations, β, Regression coefficient; RS1, Radiomic signature selected from the nodular area; RS2, Radiomic signature selected from the perinodular area; RS-C, Combined radiomic signature selected from the nodular area and perinodular area; C-R, Clinical-radiological.

**eTable 2.** Results of intra- and inter-observer intra- class correlation coefficient analysis

| Features Name | Intra-observer consistency | |  | Inter-observer agreement | |
| --- | --- | --- | --- | --- | --- |
|  | ICC | 95%CI |  | ICC | 95%CI |
| AP |  |  |  |  |  |
| original_shape_Maximum2DDiameterRow_40 | 0.951 | 0.881-0.980 |  | 0.941 | 0.877-0.972 |
| original_shape_Maximum2DDiameterSlice_40 | 0.893 | 0.751-0.956 |  | 0.822 | 0.643-0.910 |
| original_gldm_LargeDependenceEmphasis_100 | 0.974 | 0.937-0.990 |  | 0.936 | 0.863-0.969 |
| original_gldm_LargeDependenceHighGrayLevelEmphasis_100 | 0.866 | 0.693-0.945 |  | 0.881 | 0.749-0.940 |
| original_glrlm_LongRunEmphasis_100 | 0.964 | 0.912-0.985 |  | 0.923 | 0.841-0.963 |
| VP |  |  |  |  |  |
| original_firstorder_Kurtosis_40 | 0.944 | 0.866-0.977 |  | 0.934 | 0.861-0.962 |
| original_glcm_ClusterShade_40 | 0.922 | 0.813-0.968 |  | 0.899 | 0.795-0.959 |
| original_glszm_GrayLevelNonUniformity_40 | 0.954 | 0.89-0.982 |  | 0.937 | 0.868-0.973 |
| original_glszm_SizeZoneNonUniformity_40 | 0.896 | 0.756-0.957 |  | 0.825 | 0.675-0.910 |
| original_shape_SurfaceVolumeRatio_40 | 0.986 | 0.964-0.994 |  | 0.961 | 0.907-0.981 |
| original_firstorder_Skewness_100 | 0.903 | 0.773-0.961 |  | 0.889 | 0.765-0.951 |
| original_gldm_LargeDependenceHighGrayLevelEmphasis_100 | 0.758 | 0.492-0.896 |  | 0.773 | 0.558-0.885 |
| original_glrlm_RunLengthNonUniformity_100 | 0.966 | 0.916-0.986 |  | 0.941 | 0.878-0.976 |
| original_glrlm_ShortRunEmphasis_100 | 0.955 | 0.892-0.982 |  | 0.924 | 0.845-0.973 |
| original_shape_MajorAxisLength_100 | 0.991 | 0.979-0.997 |  | 0.990 | 0.975-0.999 |

Abbreviations: ICC, intraclass correlation efficient; CI, confidence interval; AP, arterial phase; VP, venous phase.

**eTable 3.** Radiomics features extraction, and selection

| Monoenergetic image Name | Total number of features | Number of features after selection method | | Finally selected features |
| --- | --- | --- | --- | --- |
|  |  | ICC | LASSO |  |
| AP |  |  |  |  |
| 40keV | 107 | 78 | 2 | original_shape_Maximum2DDiameterRow_40 |
|  |  |  |  | original_shape_Maximum2DDiameterSlice_40 |
| 100keV | 107 | 81 | 3 | original_gldm_LargeDependenceEmphasis_100 |
|  |  |  |  | original_gldm_LargeDependenceHighGrayLevelEmphasis_100 |
|  |  |  |  | original_glrlm_LongRunEmphasis_100 |
| VP |  |  |  |  |
| 40keV | 107 | 86 | 5 | original_firstorder_Kurtosis_40 |
|  |  |  |  | original_glcm_ClusterShade_40 |
|  |  |  |  | original_glszm_GrayLevelNonUniformity_40 |
|  |  |  |  | original_glszm_SizeZoneNonUniformity_40 |
|  |  |  |  | original_shape_SurfaceVolumeRatio_40 |
| 100keV | 107 | 84 | 5 | original_firstorder_Skewness_100 |
|  |  |  |  | original_gldm_LargeDependenceHighGrayLevelEmphasis_100 |
|  |  |  |  | original_glrlm_RunLengthNonUniformity_100 |
|  |  |  |  | original_glrlm_ShortRunEmphasis_100 |
|  |  |  |  | original_shape_MajorAxisLength_100 |

Abbreviations: ICC, intraclass correlation efficient; LASSO, Least Absolute Shrinkage and Selection Operator; AP, arterial phase; VP, venous phase.

**eTable 4.** Multivariate logistic regression analysis parameters of the combined model of arterial phase and venous phase

| Model | Intercept/ Signature Name | Multivariate logistic regression analysis | | |
| --- | --- | --- | --- | --- |
|  |  | β | OR（95 %CI） | P value |
| Model 4 | Intercept=0.03 |  |  |  |
|  | Clinical Signature | 0.11 | 1.12（1.07-1.17） | <0.001* |
|  | Rad-score (AP) | 0.76 | 2.14（1.57-2.93） | <0.001* |
| Model 5 | Intercept=0.01 |  |  |  |
|  | Clinical Signature | 0.10 | 1.11（1.07-1.16） | <0.001* |
|  | Rad-score (VP) | 0.79 | 2.21（1.68-2.90） | <0.001* |

Abbreviations: β, Regression coefficient; Model 4 corresponds to the clinical-radiomics combined model in arterial phase; Model 5 the clinical-radiomics combined model in venous phase; OR, Odds ratio; CI, Confidence interval; β, Regression coefficient; *, Significant at p＜0.001.
